# Hedgehog Signaling Inhibitors as Anti-Cancer Agents in Osteosarcoma

**DOI:** 10.3390/cancers7020784

**Published:** 2015-05-13

**Authors:** Ram Mohan Ram Kumar, Bruno Fuchs

**Affiliations:** Laboratory for Orthopaedic Research, Balgrist University Hospital, Sarcoma Center-UZH University of Zurich, Zurich 8008, Switzerland; E-Mail: bfuchs@balgrist.ch

**Keywords:** osteosarcoma, hedgehog signaling, GLI, SMO, hedgehog inhibitors

## Abstract

Osteosarcoma is a rare type of cancer associated with a poor clinical outcome. Even though the pathologic characteristics of OS are well established, much remains to be understood, particularly at the molecular signaling level. The molecular mechanisms of osteosarcoma progression and metastases have not yet been fully elucidated and several evolutionary signaling pathways have been found to be linked with osteosarcoma pathogenesis, especially the hedgehog signaling (Hh) pathway. The present review will outline the importance and targeting the hedgehog signaling (Hh) pathway in osteosarcoma tumor biology. Available data also suggest that aberrant Hh signaling has pro-migratory effects and leads to the development of osteoblastic osteosarcoma. Activation of Hh signaling has been observed in osteosarcoma cell lines and also in primary human osteosarcoma specimens. Emerging data suggests that interference with Hh signal transduction by inhibitors may reduce osteosarcoma cell proliferation and tumor growth thereby preventing osteosarcomagenesis. From this perspective, we outline the current state of Hh pathway inhibitors in osteosarcoma. In summary, targeting Hh signaling by inhibitors promise to increase the efficacy of osteosarcoma treatment and improve patient outcome.

## 1. Introduction

Osteosarcoma (OS) is a highly malignant bone tumor which frequently leads to patient death due to pulmonary metastasis, in spite of conventional chemotherapy and surgical excision of the primary tumor. The survival rate is estimated to be around 60%–80% in patients treated with multidrug chemotherapy and local control interventions [1]. Most conventional OS are localized to the metaphysis of the long bones adjacent to the growth plates where elongations of bones are active especially during the puberty stages. Approximately 1,000 cases of OS are reported in the USA each year and the greatest incidence rate of OS is in children and adolescents. OS arises from the malignant transformation of mesenchymal cells which differentiate towards formation of osteoid and bone. Most patients with newly diagnosed OS present with localized disease but unfortunately 15%–30% of the patients already have metastases detectable at the time of diagnosis. In OS, the most common site of metastasis is the lung which comprises more than 85% of metastatic disease, with bone being the second most common site of distant disease [2].

Apart from treatment modalities like surgery, chemo- and radiation therapy there are no effective, alternative therapies for the treatment of malignant OS and as such, the development of novel therapeutic strategies is urgently required. Aberrant activation of Hedgehog (Hh) signaling contributes to tumor aggressiveness, affecting key tumorigenic processes such as proliferation, invasion and progression of cancer cells [3]. Recently, it was shown that the Hh signaling pathway is involved in the pathogenesis of OS [4,5,6]. Therefore, inhibitors targeting Hh signaling have attracted significant attention as novel, molecularly targeted drugs. Hh signaling components including Patched (PTCH) and Smoothened (SMO) have been detected in almost 70% of OS specimens [5] and consequently, Hh signaling may play a critical role in the pathogenesis of OS. To reduce therapy side effects a desirable approach would be direct targeting of cancer signaling pathways. Translational studies are actively assessing the inhibitory activities of small molecule inhibitors of Hh pathway as promising anti-cancer agents. In this review, we summarize the development of inhibitors to treat OS, targeting the Hh signaling pathway.

### 1.1. Hh Signaling Pathway

**Figure 1:**
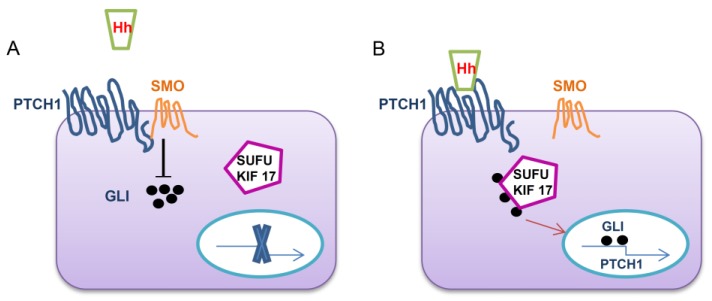
The Hedgehog signaling pathway mechanism. (**A**) In the absence of the Hh ligand, the signaling is inactive as SMO is repressed by PTCH1; (**B**) In the presence of Hh ligand it can bind to PTCH1, which relieves SMO from repression and allows downstream activation of the pathway through the translocation of GLI where it acts as a transcription factor to the nucleus with the mediation of SUFU and KIF17. Hh *Hedgehog ligand*, SMO *smoothened*, PTCH1 *patched* 1, GLI *glioma-associated oncogene family zinc finger*.

The Hh pathway acts as an organizer for embryonic development, patterning, growth control and mediates morphogenesis [7]. In recent years, the Hh signaling pathway has emerged as a critical determinant of cancer initiation, progression and metastasis of many types of cancers [8,9]. Hh pathway is necessary for early embryogenesis and eventually ceases, however, in many types of cancers, including OS, the signaling is re-activated [4,9]. Therefore, the regulation of Hh signaling in OS is important. Three ligands have been identified in Hh signaling; Sonic Hedgehog (SHh), Indian Hedgehog (IHh) and Desert Hedgehog (DHh) [7,8]. In addition, 12-transmembrane Patched proteins (PTCH1 and PTCH2), the 7-transmembrane protein, smoothened (SMO) and the 5-zinc-finger transcription factors, GLI1, GLI2 and GLI3 (glioma-associated oncogene homologs) are also identified [10,11]. Canonical Hh signaling involves the binding of one of the ligands to the transmembrane receptor PTCH1 [12]. The binding of Hh ligand in PTCH1 relieves G-protein coupled receptor-like protein SMO. After SMO is relieved by Hh ligands it leads to downstream activation of the transcription factors known as GLI family zinc finger proteins (GLI) by suppressor of fused (SUFU) and kinesin family member 17 (KIF17) [13] (Figure 1). Mutual interaction of SMO and the GLI proteins are believed to occur in the primary cilium, and it is hypothesized that cells without primary cilia cannot respond to Hh ligand through the canonical pathway [14]. In the absence of Hh ligands, PTCH inhibits SMO and rejects its entry to the cilium where it is believed to inhibit various protein kinases like PKA, GSK-3b and CK1 [15]. As a result of this, GLI proteins in complex with SUFU are phosphorylated by protein kinases which results in proteolytic cleavage where GLI-2 is degraded to repressor GLI2-R, GLI-3 is degraded to GLI3-R while GLI1 remains in full length. Typical Hh target genes are those that regulate the transcription of the Hh responsive genes by themselves which include the components of the pathway PTCH and GLI1. Other target genes include specific transcription factors such as cyclin D1 (CCND1), BMI1 polycomb ring finger (BMI1), B-cell CLL/lymphoma 2 (BCL2), and vascular endothelial growth factor (VEGF) *etc.* [16].The Hh signaling pathway is unique as most of the components consist of both oncogenes as well as tumour suppressor genes.

Of all the components of Hh signaling, GLI1 gene is a unique as it has no reported somatic mutations while other components of the pathway including PTCH, SMO, Suppressor of Fused SUFU, GLI2, and GLI3 have mutations [17,18,19,20,21,22]. However, GLI1 transcripts undergo alternative splicing leading to the synthesis of an N-terminal deletion variant (GLI1ΔN) [23] and truncated GLI1 variant (tGLI1) [24]. GLI1ΔN variant is similar to GLI1 in which it also targets the same genes of GLI1. The expression patterns of GLI1ΔN differ from that of tGLI1 in that GLI1ΔN is expressed in normal and cancerous tissues similar to GLI1 [23]. However, tGLI1 variant is expressed only in tumour cells and tissues, but undetectable in normal tissues [25]. The difference between tGLI1 variant and GLI1 is 41 amino acids and since there is only minute difference in size between them indicate the possibility that some of the previous functions attributed to GLI1 may have been due to tGLI1. tGLI1 expression influence cell motility and invasion as reported in glioblastoma and breast cancers [23,25]. tGLI1 is reported to associate with VEGF-A gene promoter leading to its activation thereby provoking angiogenesis [25]. The ability of tGLI1 to activate cancer-mediating genes like CD24, VEGF-A, MMP-2, and MMP-9 [25,26] make it more potent transcriptional regulator than GLI1 or GLI1ΔN.

In Hh signaling, mutations and loss of heterozygosity (LOH) in PTCH1, with most of the mutations in SMO are reported [27]. More than 90% of basal cell carcinomas (BCCs) harbour mutations in SMO [28]. Atwood *et al.* identified SMO mutations in 50% of resistant BCCs and showed that these mutations maintain aberrant Hh signaling even in the presence of SMO inhibitors. In some of the cancers active SMO mutant proteins fail to co-localize with PTCH1 thereby allowing the activation of the pathway independently of Hh signaling [29]. Several studies have been reported that activation of Hh signaling is also caused due to the mutations in SMO gene. Mutations in SMO are frequent in ameloblastomas of the maxilla caused by substitution of amino acid (Leu412Phe) [30]. A somatic missense mutation in SMO, caused by substitution of an amino acid in the seventh transmembrane domain (Trp535Leu), a site predicted to disrupt G-protein coupling, cause SMO activation [21]. Therapeutic challenges remain where tumors acquire resistance to SMO antagonists, and also in cases where signaling is driven by active SMO mutants that exhibit reduced sensitivity to these compounds.

### 1.2. Hh Signaling and Its Induction of Malignancy in Osteosarcoma

Several evolutionary signaling pathways, such as Hh, Notch, Wnt and BMP-TGF beta-activin are involved in the proper development of normal bone. It is also becoming increasingly clear that these pathways can have a crucial role in many types of cancer. Of those signaling pathways, most of the studies are now focused on Hh signaling in OS pathogenesis [31], rendering the inhibition of this pathway as an interesting approach to control disease progression. Mohseny *et al.* reported that activation of Hh pathway varied among various OS cell lines analysed and did not correlate with the patient survival [32]. However, Lo *et al.* analysed Hh pathway genes in 43 human primary high-grade OS samples and determined that expression levels of genes encoding IHH, PTCH1 and GLI genes but not SMO were higher in tumour specimens [5]. Ligand-dependent activation (IHH-PTCH1 co-expression) and ligand independent (SMO, PTCH1, GLI) might lead to Hh stimulation in OS. Presently, this ligand-dependent pathway is thought to be the major mechanism underlying Hh signaling activation. Moreover, the high levels of IHh may lead to larger tumor size, a prognostic factor of OS thereby indicating that activation of Hh signaling is required for OS progression [5]. Among the Hh components, recent studies have shown that SMO and GLI activation are important components in the progression of OS. Hirotsu *et al.* analysed the transcripts of Hh genes in OS cell lines (NHOst, 143B, HOS, MG63 and NOS-1) and determined that SHh, DHh, PTCH1, SMO, GLI1 and GLI2 were overexpressed. However, only SMO, PTCH1, and GLI2 transcripts were over-expressed in human OS biopsy specimens [4]. One of the interesting observations was the downregulation of GLI1 and upregulation of GLI2 in human OS biopsy specimens. The authors hypothesized that the GLI1 promoter is inactivated in human OS by epigenetic modification and that Hh pathway activity downstream of SMO is mediated only by GLI2. A recent article by Kitamoto *et al.* showed that the high expression levels of GLI2 correlated with lung metastasis and has poor clinical outcome in mice [33] but there was no correlation between the location of the OS and GLI2 expression. Since the sample size used in the study was low the relationship between GLI2 expression and prognosis could not be determined. Nagao *et al.* demonstrated that GLI2 is involved in the migration, invasion and metastasis by regulating the cell cycle genes *in vitro* [8]. The importance of Hh signaling in OS was further revealed from the studies on knockdown of GLI2 in nude mice. Inoculation of 143B OS cells transfected with GLI2- shRNA resulted in a significant reduction of tumour growth as compared with inoculation of 143B cells transfected with control shRNA. The silencing of GLI2 in nude mice provided a statistically significant survival benefit [8]. Treatment using GLI-2 shRNA approach might be a promising. Expression of GLI2 also correlates with poor outcome in OS patients and siRNA silencing of GLI2 increased the sensitivity of OS cell lines to chemotherapeutic drugs [34]. Moreover, microarray expression profiles of normal, metastatic and non-metastatic OS patient samples further revealed that SMO was expressed highly in metastatic OS [35]. Silencing of SMO by SMO shRNA inhibited OS growth in xenograft mouse models and conferred a significant survival benefit [4]. Interaction between Hh signaling components and other pathways have to be studied in more detail as aberrant Hh signaling cannot alone initiate OS progression. Chan *et al.* generated an osteoblastic OS mouse model and found that upregulated Hh signaling interacts with Yap1, the main effector of the Hippo tumor suppressor pathway and H19, a long non-coding RNA. Since YAP1 expression is also upregulated in human OS patient specimens, inhibition of Hh signaling and YAP1 together could present a significant therapeutic value [36]. More evidence on mechanistic details of Hh signaling in OS are not discussed here in order to focus on Hh signaling inhibitors in OS.

### 1.3. Hedgehog Signaling Inhibitors in Osteosarcoma

#### 1.3.1. SMO Inhibitors

In recent years, Hh pathway drug discovery has been focused predominantly on targeting SMO, central transducer of the Hh signaling. SMO is widely considered as the most druggable target in the Hh signaling pathway. SMO inhibition alters transcription factors GLI1 and GLI2 to remain inactive, which prevents the expression of tumor mediating genes within the Hh pathway. A recent review on Hh inhibitors indicates the diversity of potential agents that are able to modulate the function of SMO. The chemical action of some of the small molecule Hh pathway inhibitors has been described in detail [37].

##### Cyclopamine

The plant-derived compound cyclopamine first emerged as a potential drug that binds to SMO and inhibits signal transduction to the nuclear target gene GLI. Warzecha *et al.* concluded that because of limited potency and remarkable side effects of cyclopamine in OS it was not suitable for clinical development [38]. Also, there was no significant difference in the pulmonary metastases in the cyclopamine-treated mice group and the control group. Interestingly, studies by Hirotsu *et al.* showed that cyclopamine exhibited greater potent effect in inhibiting proliferation via cell cycle regulation *in vitro*. Cyclopamine decreased tumor growth *in vivo* but had side effects where mice experienced loss of body weight and dehydration and eventually died [4]. Moreover, cyclopamine is chemically unstable with poor solubility features [39]. These results in a mouse xenograft OS model system suggest that cyclopamine may not be a better therapeutic option for OS.

##### IPI-926 (Saridegib)

Saridegib is an oral, semi-synthetic derivative of the alkaloid cyclopamine and has demonstrated to exhibit potent anti-cancer activity in multiple preclinical animal models of cancer especially chondrosarcoma [40,41]. There is only one study that has been reported to determine the clinical efficacy of saridegib in OS. Saridegib treatment in patient-derived xenografts (PDXs), generated from tumors of patients with metastasis at diagnosis, reduced tumour growth by inhibiting the ligand-dependent Hh signaling pathway [6]. One of the limitations of this study was the small sample size and the presence of tumor heterogeneity. A larger cohort would be required to determine the efficacy of this drug in OS.

##### GDC-0449 (Vismodegib)

Vismodegib, a second generation cyclopamine, is a 2-arylpyridine molecule that blocks the activation of SMO. Considerable advantages over cyclopamine have been observed with better aqueous and acid stability. It is the first drug approved by the Food and Drug Administration (FDA) for the treatment of locally advanced basal cell carcinoma (BCC) that cannot be removed by either surgical resection or treated with radiation [42]. There is an ongoing Phase I and II clinical trial by the National Cancer Institute to determine the side effects and optimal dose of vismodegib in extra-skeletal and metastatic OS (ClinicalTrials.gov Identifier: NCT01154452). Kitamoto *et al.* showed that combination therapy of vismodegib, ATO (arsenic trioxide) and GANT61 (GLI inhibitor) at low concentrations prevented migration and metastasis of OS cells. Moreover, ATO and vismodegib significantly inhibited the metastasis of OS to the lung which suggested that the combined administration of ATO and vismodegib in xenografts might be effective for the treatment of OS metastasis [33].

##### LDE225 (Erismodegib)

Erismodegib, a SMO antagonist, induces G1 cell cycle arrest and apoptosis in many of the cancers [43]. There is an ongoing clinical trial to determine the efficacy of this drug in OS (ClinicalTrials.gov Identifier: NCT01154452) in a pediatric phase I study.

#### 1.3.2. GLI Inhibitors

GLI transcription factors are critical mediators of the Hh signaling pathway and their aberrant activation might induce tumour cell proliferation and invasion. The Hedgehog-GLI signaling pathway is active in many cancers and is known to contribute to the growth and survival of human OS cells [8]. The expression of GLI2 has been shown to be highly elevated in OS patients and that GLI2 could be considered as a new therapeutic target for OS metastasis [34]. The following are the GLI inhibitors which are found to be effective in controlling OS.

##### Arsenic trioxide (ATO)

ATO (As_2_O_3_) is an FDA-approved drug used for the treatment of patients with acute promyelocytic leukemia (APL) [44]. ATO directly binds to GLI1 and GLI2 and inhibits its transcriptional activity and deceases the expression of endogenous GLI target genes [45]. Tumour caused by intradermal inoculation of 143B cells in nude mice were significantly reduced on daily treatment with ATO and provided a significant survival benefit. Moreover, the number of apoptotic cells was also significantly increased in ATO-treated tumors [46]. Li *et al.* used ATO which was incorporated in the magnetic nanoparticles and encapsulated by poly lactic acid *in vivo* where MG63 OS cell lines were s.c injected into the mice. Tumour volume was significantly decreased in the nanoparticle encapsulated ATO treated group and the inhibitory effect was similar to those treated with ATO alone [47]. Phase II trials have been completed to determine the efficacy of this drug in OS (ClinicalTrials.gov Identifier: NCT00024258). Chiu *et al.* demonstrated that combined treatment of radiation and ATO resulted in the increased induction of autophagy and apoptosis via inhibition of the PI3K/Akt signaling pathway in OS cell lines [48]. *In vitro* studies using ATO in OS cell lines showed it inhibited migration and invasion by inactivating the MAPK/ERK signaling pathway [49]. Taken together, ATO seems to be a promising drug for treatment of OS but its effect on lung metastasis remains to be studied.

##### GANT

GLI activators Antagonists (GANT) which inhibits GLI-mediated transcription were discovered in a screen-based assay [50]. Two Gli inhibitors which have been described are GANT 61 [51] and GANT 58 [52]. GANT 61 is one of the widely studied GLI inhibitor in OS [8]. The mechanism by which GANT 61 and GANT 58 inhibit the Hh pathway at the GLI level is unknown. Current evidence suggests that GANT 61 modifies GLI 1 and GLI 2 and prevents them from binding to the DNA promoter [50]. Studies on canine derived OS cells treated with GANT61 revealed an inhibition of cell proliferation and reduced colony formation [53]. GLI2 has been reported to express highly in invasive OS cell lines but not in non-invasive cells [54], hence targeting GLI2 with GANT 61 might be strategically possible. Studies on treatment with GANT 61 in human OS cell lines revealed that GANT61 dose-dependently inhibited proliferation but the growth of tumour remained unaffected on GANT 61 treatment group when compared to mice who did not receive the drug [8]. Since GLI 2 is involved in bone development [55,56], possible side effects for using GANT 61 in therapy may include bone defects, especially in children. Currently, no clinical trials are being performed to test the efficacy of GANT61 in OS patients.

## 2. Conclusions

In this review, we have summarized the involvement of Hh signaling and its inhibitors for potential therapy in OS. The Hh signaling pathway might represent a valid therapeutic target in OS. Further, this pathway is activated in the progression of the disease and both ligand-dependent and independent inhibitors are effective. Hh signaling inhibitors are found to be effective in cancers like BCC and meduloblastoma where Hh signaling is constitutively activated and administration of Hh inhibitors as a single agent in those cancers are already used as first line therapy. Within the different classes of Hh inhibitors, recent drug development has focused on SMO inhibitors in controlling OS progression. Despite the fact that a lot of Hh pathway inhibitors have been developed, none of them has yet successfully reached the clinic for the treatment of OS. However, appropriate combination therapy with other signaling inhibitors might be required for clinical trials since the activated Hh pathway is not the primary driver of malignancy. Hh inhibitors have a potential to be a promising drug option, but there is still much to be learn about the biological effects of Hh pathway inhibition in order to explore the therapeutic potential of targeting the Hh signaling pathway in OS.
